# Analysis of homodimer formation in 12-oxophytodienoate reductase 3 *in solutio* and *crystallo* challenges the physiological role of the dimer

**DOI:** 10.1038/s41598-024-69160-6

**Published:** 2024-08-05

**Authors:** Bianca Kerschbaumer, Peter Macheroux, Aleksandar Bijelic

**Affiliations:** Institute of Biochemistry, Graz University of Technology, Petersgasse 12/II, 8010 Graz, Austria

**Keywords:** 12-oxophytodienoate reductase 3, Old Yellow Enzymes, Ene-reductases, Dimerization, Self-inhibition, Biochemistry, Structural biology

## Abstract

12-oxophytodienoate reductase 3 (OPR3) is a key enzyme in the biosynthesis of jasmonoyl-*L*-isoleucine, the receptor-active form of jasmonic acid and crucial signaling molecule in plant defense. OPR3 was initially crystallized as a self-inhibitory dimer, implying that homodimerization regulates enzymatic activity in response to biotic and abiotic stresses. Since a sulfate ion is bound to Y364, mimicking a phosphorylated tyrosine, it was suggested that dimer formation might be controlled by reversible phosphorylation of Y364 in vivo. To investigate OPR3 homodimerization and its potential physiological role in more detail, we performed analytical gel filtration and dynamic light scattering on wild-type OPR3 and three variants (R283D, R283E, and Y364P). The experiments revealed a rapid and highly sensitive monomer–dimer equilibrium for all OPR3 constructs. We crystallized all constructs with and without sulfate to examine its effect on the dimerization process and whether reversible phosphorylation of Y364 triggers homodimerization in vivo. All OPR3 constructs crystallized in their monomeric and dimeric forms independent of the presence of sulfate. Even variant Y364P, lacking the putative phosphorylation site, was crystallized as a self-inhibitory homodimer, indicating that Y364 is not required for dimerization. Generally, the homodimer is relatively weak, and our results raise doubts about its physiological role in regulating jasmonate biosynthesis.

## Introduction

**Figure 1:**
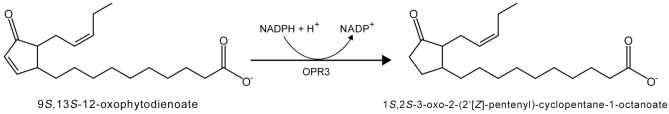
General scheme of the reaction catalyzed by 12-oxophytodienoate reductase 3 (OPR3).

12-oxophytodienoate reductase 3 (OPR3), a member of the Old Yellow Enzyme (OYE) family, is a key enzyme in the octadecanoid pathway for jasmonic acid biosynthesis by catalyzing the reduction of (9*S*,13*S*)-12-oxophytodienoic acid to the corresponding cyclopentanone (Fig. 1)^1–3^.

Jasmonates are phytohormones that are pivotal in mediating plant responses to various biotic and abiotic stresses and regulating plant growth^4^. By regulating the production of the receptor-active conjugate jasmonoyl-*L*-isoleucine, OPR3 stands at the crossroads of plant defense, developmental, and environmental adaptation processes^5^. Disruption of the *opr3* gene results in a jasmonic acid-deficient phenotype in Arabidopsis and tomato, demonstrating that OPR3 is the isozyme responsible for jasmonic acid biosynthesis, as other OPR isozymes failed to compensate for OPR3 function^6–8^.

The first crystal structure of OPR3 from tomato (*Sl*OPR3) showed that the enzyme crystallized as a self-inhibitory dimer in which the L6 loop, a critical loop involved in substrate recognition and coenzyme binding, from each protomer intrudes into the active site cavity of the other protomer^9,10^. Furthermore, it was shown that the *Sl*OPR3 variant E291K, which does not form the inhibitory dimer *in crystallo*, had a six times lower turnover than the wild type. Based on these observations, it was postulated that the formation of the self-inhibitory homodimer represents a mechanism for regulating OPR3 activity and, thereby, jasmonic acid biosynthesis. However, the homodimer is not highly stable, as evidenced by a relatively high dissociation constant of approximately 30 µM, indicating a rapid monomer–dimer equilibrium^9^. The crystal structure of *Sl*OPR3 also showed that the dimer interface is stabilized by a bound sulfate ion, which crosslinks the dimer's protomers. The sulfate ion interacts, among other residues, with Y364 in a geometry reminiscent of a phosphorylated tyrosine. Therefore, it was suggested that homodimerization could be regulated by reversible phosphorylation of Y364 in vivo. We recently showed that Y364 also interacts with the 2’-phosphate group of the coenzyme NADPH, concluding that it is part of the coenzyme-phosphate binding site comprising the residues R343, Y364, and R366.

Physiologically relevant self-inhibitory dimerization is relatively rare among enzymes, although some examples exist, including *Clostridium botulinum* neurotoxin serotype A and the TATA-binding protein^11,12^. Despite the prevailing theory that OPR3 dimerization serves as a modulator of enzymatic activity in vivo, the weak nature of the *Sl*OPR3 dimer and the scarcity of such dimers, in general, raise doubts about the proposed physiological link between OPR3 dimerization and activity regulation.

Therefore, we aimed to critically examine OPR3 dimerization as a requisite mechanism for activity regulation. We investigated the dimerization process of wild-type *Sl*OPR3 and three variants (R283D, R283E, and Y364P) in solution by analytical gel filtration and dynamic light scattering (DLS). We then subjected the enzymes to X-ray crystallography to structurally characterize dimer formation, demonstrating that *Sl*OPR3 dimerization is highly dynamic and structurally diverse and that Y364 is not required for homodimerization.

## Results and discussion

### Homodimerization in solution

To better understand the behavior of *Sl*OPR3 in solution, we performed analytical gel filtration and DLS with the wild-type enzyme and the variants R283D, R283E, and Y364P. The variants R283D and R283E were produced because residue R283 was shown to play an important role in substrate and coenzyme binding, so we aimed to test whether this residue also affects the enzyme's dimerization process^10,13^. Variant Y364P was produced to test whether residue Y364 is required for homodimerization, as proposed previously^9^. Proline was chosen because other well-studied ene-reductases, such as morphinone reductase, contain a proline at the corresponding position to Y364.

#### Analytical gel filtration

At a concentration of 2 mg/ml, which is slightly above the previously determined dissociation constant of the self-inhibitory dimer (30 µM ≈ 1.4 mg/ml), all enzymes eluted mostly in their monomeric state (see Supplementary Fig. S1). While the wild type and Y364P almost exclusively eluted in their monomeric form, R283D and R283E showed some peak broadening at the base, indicating some self-association.

Even at the highest applied concentrations (10–20 mg/ml) at which the column was overloaded, the enzymes eluted mainly in their monomeric form, indicating weak or transient homodimerization under the given conditions (see Supplementary Fig. S1). The resolution of the used column was sufficient to separate the monomeric (44.6 kDa) from the dimeric state (89.2 kDa); thus, we exclude the possibility that a large homodimer fraction eluted together with the monomeric form as a single peak.

Our results somewhat contrast those previously reported, where the authors observed a clear shift to higher molecular weights with increasing protein concentrations^9^. Gel filtration might not detect transient or weak dimers if the monomer–dimer equilibrium shifts rapidly. Accordingly, under different conditions (e.g., buffer composition), the dimer might be more or less stabilized and thus detectable by gel filtration, explaining the difference between our and the previous results.

Regarding the variants, the results show that substituting residues R283 and Y364 had no significant effect on the enzyme's behavior during gel filtration.

#### Dynamic light scattering (DLS)

To better capture dynamic associations in solution, we performed DLS. DLS was carried out in the presence and absence of 25 mM ammonium sulfate to investigate the effect of Y364-bound sulfate, which may mimic phosphorylated Y364, as previously suggested^9^. For comparison, the theoretical hydrodynamic diameters for the monomeric and dimeric forms of all enzymes were computationally calculated (wild type: 5.58 and 9.54 nm; R283D: 5.58 and 9.43 nm; R283E: 5.58 and 9.35 nm; Y364P: 5.58 and 9.52 nm).

**Figure 2:**
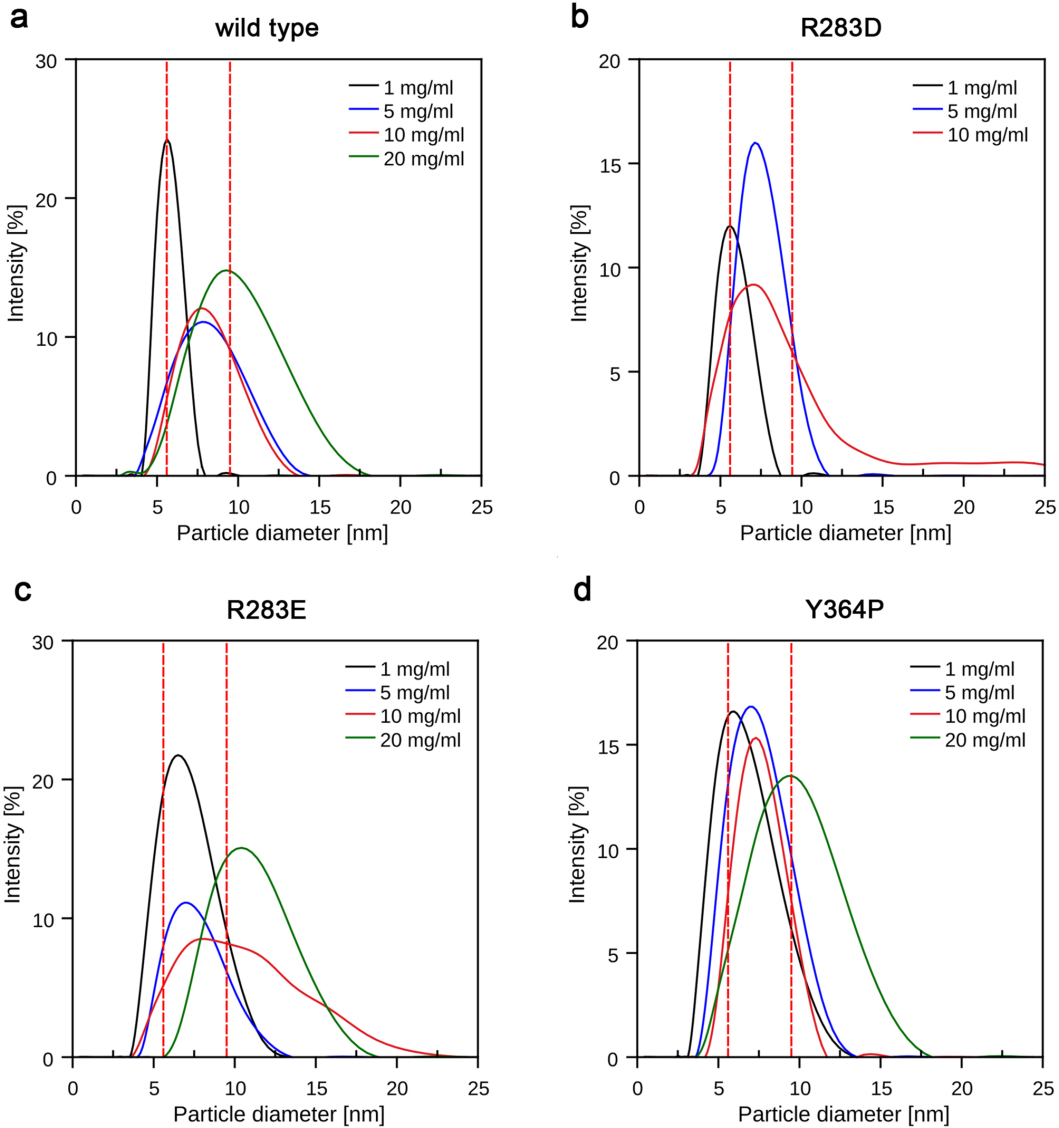
Dynamic light scattering (DLS) profiles. DLS results for *Sl*OPR3 (**a**) wild type, (**b**) R283D, (**c**) R283E, and (**d**) Y364P. R283D was not completely soluble at a concentration of 20 mg/ml. The red vertical dashed lines indicate the calculated particle diameters for the enzymes' monomeric and dimeric states.

In contrast to analytical gel filtration, DLS showed a clear concentration-dependent shift of the hydrodynamic diameter toward larger sizes for all enzymes (Fig. 2). At the lowest tested concentration (1 mg/ml), all enzymes showed a sharp peak at a particle size of ca. 5–6 nm, which corresponds well to the calculated diameter of the monomeric enzyme (5.58 nm). Furthermore, the polydispersity index was < 0.3 in most cases, indicating a relatively homogenous solution. At higher concentrations (5 and 10 mg/ml), the samples were polydisperse (polydispersity index > 0.3), and the particle size shifted toward values between 6 and 8 nm, indicating a rapid equilibrium between the monomeric and dimeric forms. R283E showed the strongest tendency toward the monomeric state among the variants, as even at a concentration of 5 mg/ml, it exhibited mainly the monomeric state. At the highest tested concentration (20 mg/ml), all samples, except for R283D, which was not completely soluble at this concentration, exhibited a hydrodynamic diameter of around 10 nm, agreeing well with the calculated values of 9.18–9.54 nm for the respective dimer. Interestingly, variant Y364P, which, because of the lack of Y364, was not expected to dimerize readily, showed a clear tendency for dimerization with increasing concentration.

The addition of ammonium sulfate had no significant effect (*p* > 0.05) on the diameter size (see Supplementary Figure S2). Thus, sulfate binding to Y364 did not promote dimerization in the Y364-bearing constructs. In general, adding ammonium sulfate increased the solubility of the enzymes, which was particularly evident for R283D, as the variant was only in the presence of sulfate soluble at 20 mg/ml.

Our DLS experiments confirmed the concentration-dependent and highly dynamic nature of OPR3 dimerization in vitro. Ammonium sulfate failed to promote homodimerization, arguing against the previously suggested importance of sulfate-mediated interactions for dimerization. DLS showed a substantially higher dimer proportion at the highest tested protein concentration than analytical gel filtration, indicating that the monomer–dimer equilibrium was too rapid to be captured in real-time by the latter technique, which is less sensitive than DLS.

### X-ray crystallography

We crystallized the *Sl*OPR3 wild type and variants, trying to capture each enzyme in its monomeric and dimeric state to provide structural insights into the dimerization process of *Sl*OPR3. All enzymes were successfully crystallized in their monomeric and dimeric forms using either the same or similar crystallization conditions. In some cases, crystals of the monomeric and dimeric forms were even obtained from the same crystallization drop, corroborating that both forms exist in an equilibrium in solution.

#### Sl*OPR3 also crystallizes in its monomeric form*

**Figure 3:**
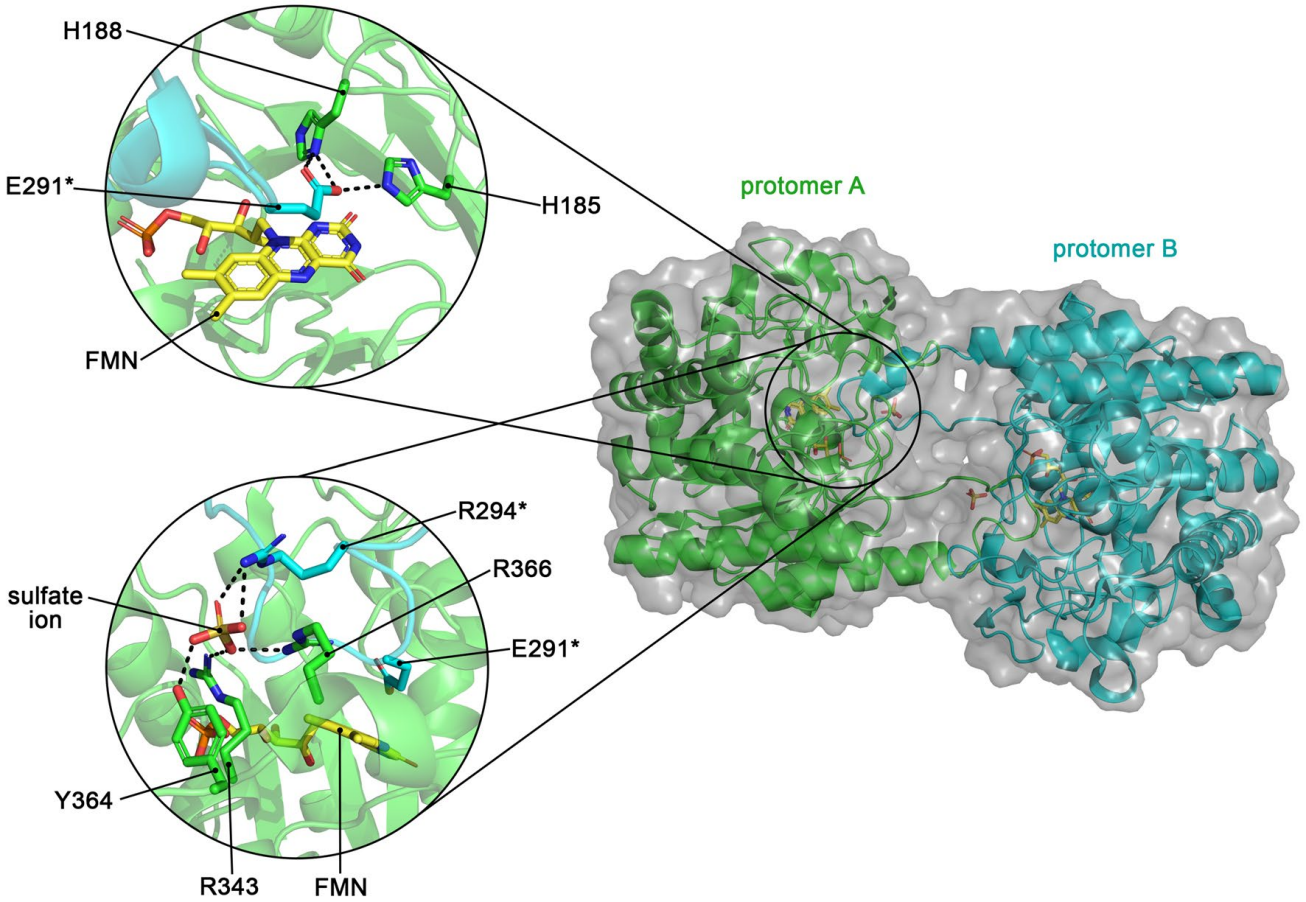
Crystal structure of the self-inhibitory dimer of wild-type *Sl*OPR3 (PDB entry: 2HSA). The left upper inset shows how residue E291* from the intruding protomer (cyan cartoon and sticks) is placed above the FMN molecule of the other protomer (green cartoon and sticks), interacting with the catalytic residues H185 and H188. E291* is located where the electron-withdrawing groups of *Sl*OPR3 substrates bind. The left lower inset shows the sulfate ion-mediated dimer interface, where the sulfate ion interacts with R343, Y364, and R366 from one protomer (green cartoon and sticks) and R294* from the other one (cyan cartoon and sticks). Note the interaction between the sulfate ion and Y364, which putatively mimics a phosphorylated tyrosine. Asterisks* indicate residues from a different protomer; black dashed lines indicate H-bonds and electrostatic interactions.

Wild-type *Sl*OPR3 was initially crystallized as a self-inhibitory homodimer in which the L6 loop from each protomer intrudes into the active site cavity of the other protomer, placing residue E291 above each other's flavin cofactor and thereby inhibiting the enzyme (Fig. 3). Using the original crystallization conditions, including ammonium sulfate, we obtained only the self-inhibitory dimer. The bound sulfate ion was found to be a structural hallmark, as it seems required for dimer stabilization by interacting with R343, Y364, and R366 from one protomer and with R294 from the other (Fig. 3, inset). The sulfate ion is located close to Y364, mimicking a phosphorylated tyrosine residue, which is why it was suggested that homodimerization, and thus OPR3 activity, might be regulated by reversible phosphorylation of Y364 in vivo.

To test this hypothesis, we crystallized wild-type *Sl*OPR3 without ammonium sulfate. We found a condition that produced high-quality crystals without sulfate, using sodium tartrate as an additive instead. Most of these crystals contained the enzyme in its monomeric state when crystallized at a protein concentration of 10 mg/ml. The crystal structure of the monomeric wild type (PDB entry: 9EM3) is almost identical to that of the single protomers in PDB entry 2HSA (RMSD_Cα_ = 0.27 Å) but lacks loop L6 almost completely due to disorder, demonstrating the flexible nature of this loop.

At higher concentrations (20 mg/ml), only the self-inhibitory dimer was obtained, confirming the protein concentration dependency of homodimerization. In the homodimer structure obtained without ammonium sulfate (PDB entry: 9EM2), only one protomer has a bound tartrate molecule at the site where the sulfate ion usually binds. The tartrate molecule, as well as the sulfate ion in the original homodimer structure, is mainly bound by the positively charged arginine residues R343 and R366, with Y364 only playing an assisting role by providing an additional H-bond.

These results demonstrate that 'sulfurization' of Y364—and consequently the proposed phosphorylation—is not required for dimerization. In a recent study, we also showed that the binding site at which the sulfate ion is bound is involved in NADPH binding^10^. Therefore, we conclude that one of the site's functions is binding the 2’-phosphate group of NADPH.

While collecting data for our previous study^10^, we observed that in the presence of micromolar concentrations of weakly binding ligands (e.g., NADH; dissociation constant = 1.4 mM), the enzyme crystallized exclusively in its monomeric state, indicating that the monomer–dimer equilibrium is readily modulated, which also argues against a physiological role of the homodimer. The weak nature of the self-inhibitory dimer does not necessarily disprove the proposed regulatory role but adds complexity to this regulation mechanism.

#### Sl*OPR3 dimerization is dynamic*

**Figure 4:**
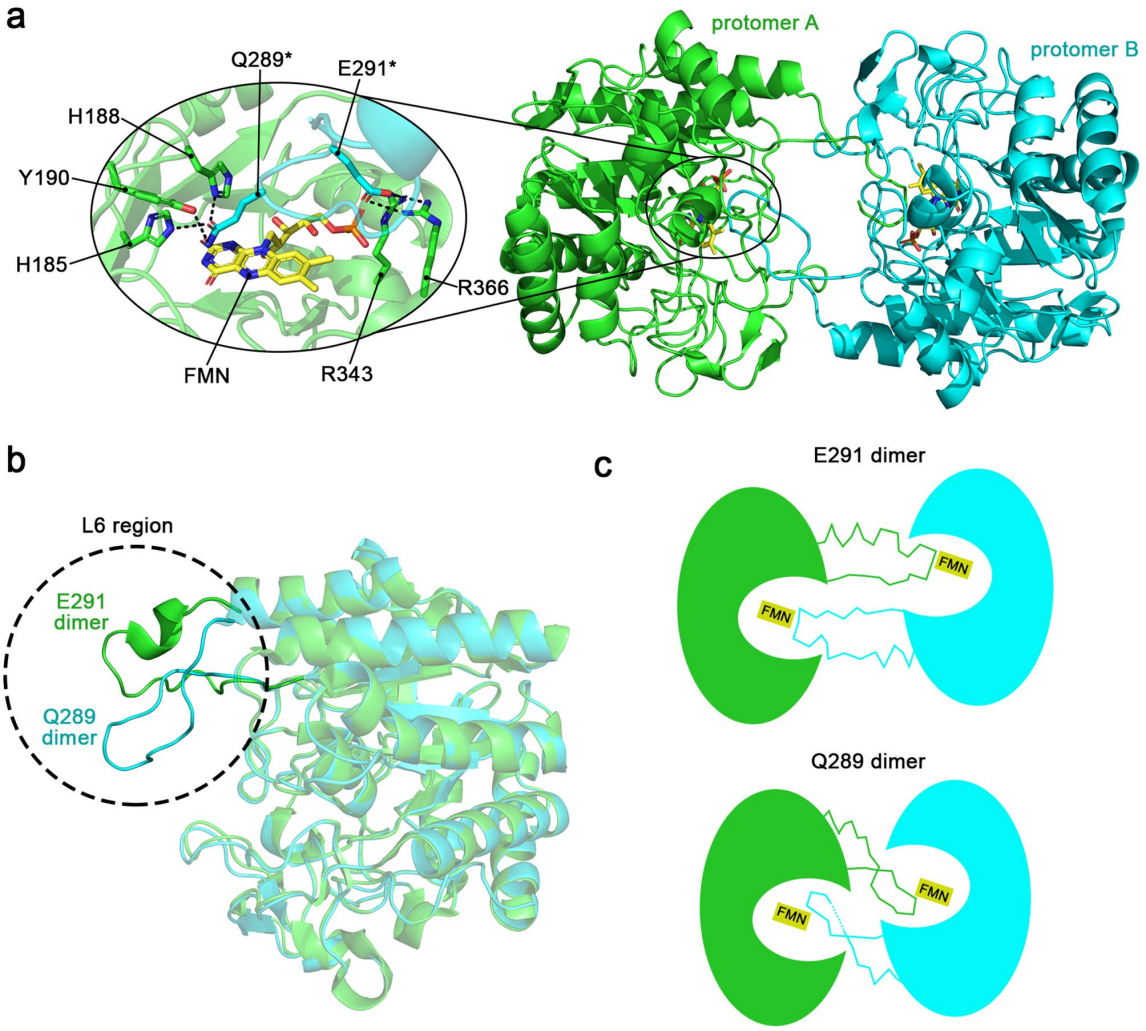
Crystal structure of the novel wild-type *Sl*OPR3 self-inhibitory dimer. (**a**) Overall structure of the novel wild-type *Sl*OPR3 homodimer. The left inset shows the interactions of Q289* and E291* from one protomer (cyan cartoon and sticks) with residues of the other protomer (green cartoons and sticks). Asterisks* indicate residues from a different protomer; black dashed lines indicate H-bonds and electrostatic interactions. (**b**) Superimposition of one protomer from the initially reported homodimer (PDB entry: 2HSA; green cartoon and denoted as E291 dimer) onto that from the here reported novel homodimer (PDB entry: 9EM0; cyan cartoon and denoted as Q289 dimer). The dashed circle highlights the differences in the L6 region. (**c**) Schematic comparison of the original (E291 dimer) and the novel homodimer (Q289 dimer).

During this study, we analyzed multiple data sets of the wild-type homodimer. Interestingly, the homodimers were not always highly similar or identical, exhibiting RMSD_Cα_ values ranging between 1 and 4 Å. In most cases, the homodimers differed in the conformation of their L6 loops and the position and orientation of the single protomers relative to each other. One self-inhibitory dimer (PDB entry: 9EM0) was markedly different from the reported one. In this dimer, the L6 loops do not adopt a stretched conformation like in the original self-inhibitory dimer (PDB entry: 2HSA) but a bent and twisted conformation, leading to Q289 being located above the flavin cofactor instead of E291 (Fig. 4).

In contrast to E291 in the original homodimer structure, Q289 does not only H-bond to the catalytic residues H185 and H188 but also to the proton donor Y190 (Fig. 4A, inset). While Q289 is located above the flavin in this novel wild-type homodimer, E291 contributes strongly to the dimer interface by electrostatically interacting with R343 and R366 from the other protomer. In the original homodimer, Q289 is not involved in any significant interactions.

The substantial difference in the L6 conformations between the original and the novel wild-type dimer leads to a more compact dimer for the latter (hydrodynamic radius: 9.54 nm vs. 9.18 nm; Fig. 4B and C).

These results show that the homodimer of wild-type *Sl*OPR3 exhibits conformational variability, further questioning the proposed physiological role of the self-inhibitory homodimer.

#### Variant R283E crystallizes primarily in its monomeric form

Variant R283E crystallized readily in its monomeric form (PDB entry: 8QN9) under the same conditions (including ammonium sulfate) used to crystallize the homodimeric wild type. The crystal structure of R283E is almost identical to that of the wild type (RMS_Cα_ = 0.22 Å), except for the R283E substitution and L6, which lacks 16 amino acids (V285–E301) due to disorder. As R283E initially failed to form a homodimer *in crystallo*, we assumed that the amino acid exchange disrupted dimer-forming and -stabilizing interactions. However, our DLS results showed that at higher concentrations, the monomer–dimer equilibrium is clearly shifted toward the dimer.

**Figure 5:**
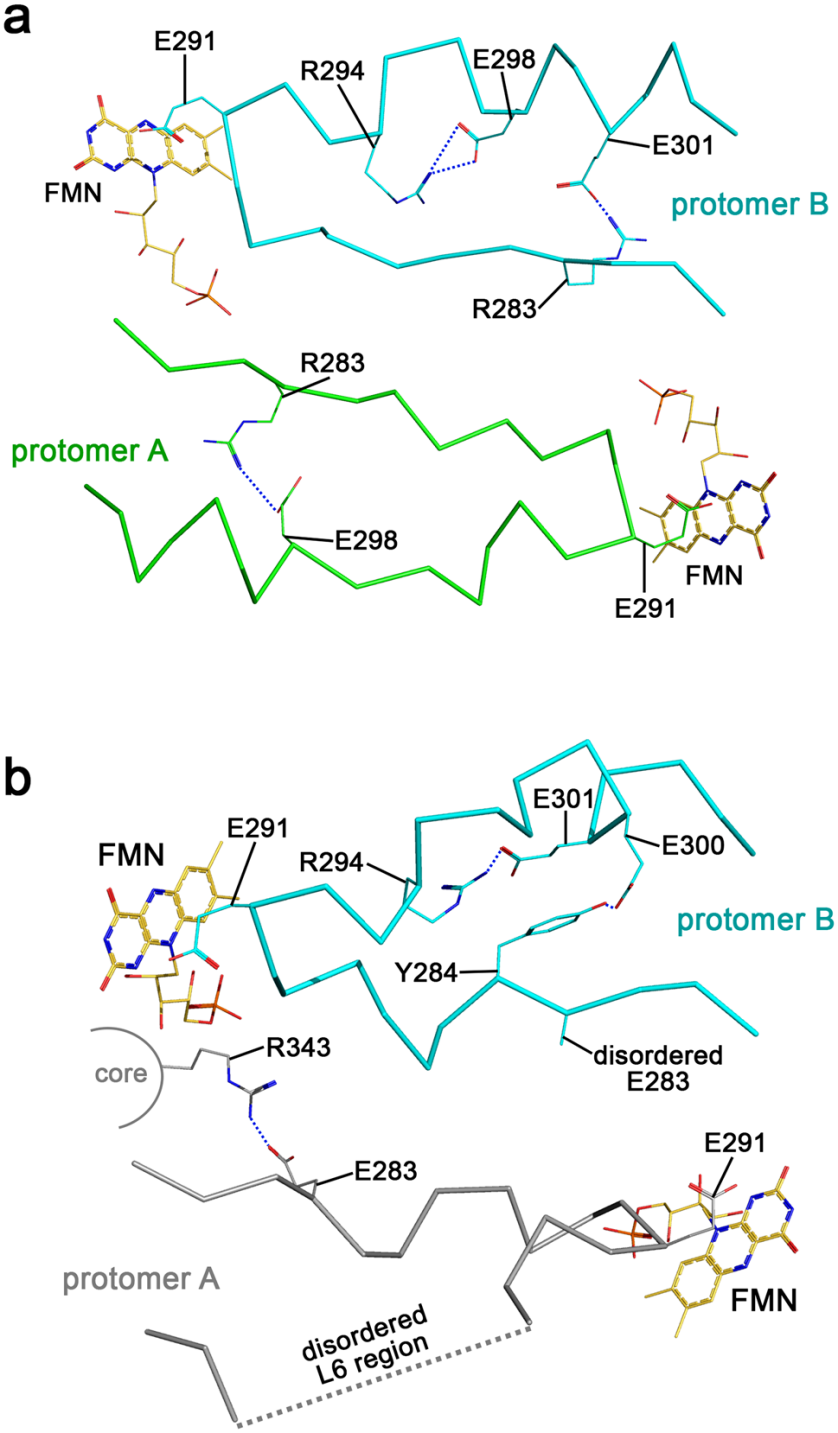
Comparison of the dimer-forming L6 loops between *Sl*OPR3 wild-type and R283E. (**a**) L6 conformations in the wild-type homodimer. (**b**) L6 conformations in the R283E dimer; the dotted line indicates the disordered region of L6 for which no electron density was observed. The loops are depicted as ribbons while interacting amino acid residues are shown as lines. FMN molecules are also shown for orientation. Blue dashed lines indicate H-bond and electrostatic interactions. Note the lack of intramolecular salt bridges within L6 of protomer A in R283E.

We ultimately crystallized R283E in its homodimeric form (PDB entry: 8S8Y) using almost the same conditions as for the monomeric form (only the PEG concentration differed). The wild-type self-inhibitory and R283E homodimer differ mainly in their L6 conformations (Fig. 5). The sulfate binding site of both homodimers superimposes well, but no sulfate ion is bound in the R283E-dimer structure, corroborating that the sulfate-mediated interactions are not required for dimer formation.

The dimer interface in R283E seems less stable than that in the wild type because loop L6 of one protomer in R283E is highly flexible, as evidenced by the quality of the electron density map.

So, substituting R283 for glutamic acid seems to destabilize loop L6 and thus the dimer. The reason why E283 destabilizes the dimer is not immediately apparent. A precise comparison of the dimers suggests that the amino acid substitution disrupts interactions that stabilize the stretched conformation of L6. In the wild-type homodimer, R283 is involved in intramolecular salt bridges that—together with other salt bridges—seem to maintain the stretched conformation of both loops in the dimer (Fig. 5, top). These R283-mediated salt bridges are missing in R283E. Instead, E283 forms a salt bridge with R343 in one protomer, which is located at the enzyme's active site entrance, thereby destabilizing the whole loop L6, as evidenced by missing electron density for large parts of the loop (Fig. 5, bottom). However, in the second protomer, the stretched conformation of L6 is maintained, as a salt bridge between R294 and E301 and a strong H-bond between Y284 and E300 compensate for the missing R283-mediated salt bridge; E283 is not resolved in this protomer due to disorder.

Considering both the DLS and crystallographic data, it seems that the amino acid substitution R283E increases the flexibility of loop L6 in the corresponding variant, impeding proper dimer formation.

#### Variant R283D forms a *pseudo*-semi-inhibitory homodimer

**Figure 6:**
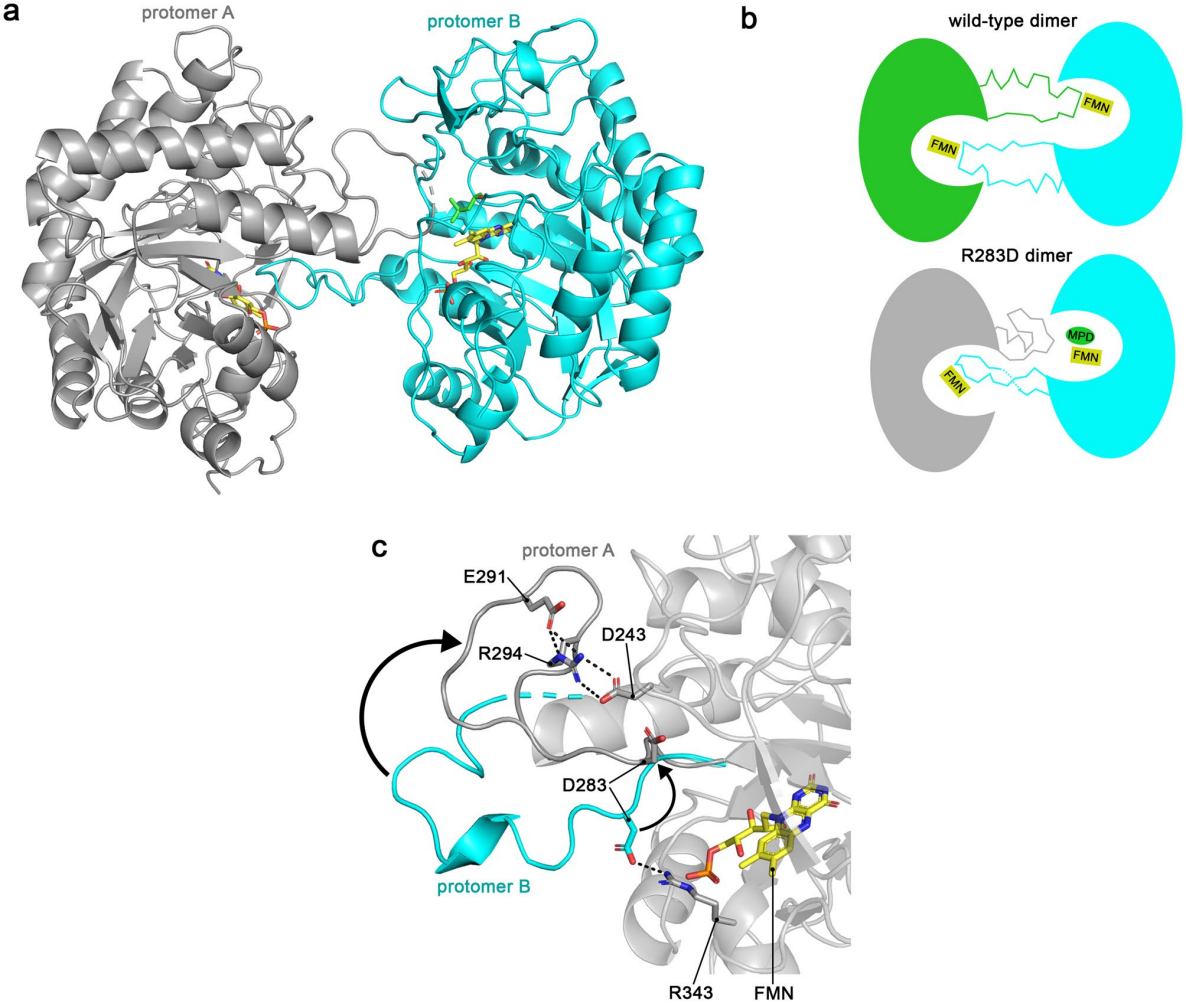
Crystal structure of the semi-self-inhibitory dimer of R283D. (**a**) Overall structure of the semi-self-inhibitory R283D dimer. (**b**) Schematic comparison of the original homodimer (wild-type dimer) and the semi-inhibitory homodimer (R283D dimer). (**c**) Superimposition of the L6 loops of protomer A (grey cartoon and sticks) and protomer B (cyan cartoon and sticks) of R283D. In protomer B, D283 is electrostatically locked by R343, while in protomer A, it can freely move. Black arrows indicate the movement of L6 to transition from the stretched into the folded conformation. Black dashed lines indicate H-bond and electrostatic interactions.

In contrast to R283E, variant R283D tended to crystallize in its dimeric form (PDB entry: 8QN1). The self-inhibitory dimer of variant R283D differs from that of the wild type, as only loop L6 of one protomer intrudes into the active site of the other protomer, while the other loop only covers the active site entrance of the other protomer (Fig. 6A and B). As a result, the active site of only one protomer is entirely blocked while that of the other protomer is still accessible to at least small molecules, as evidenced by a bound MPD molecule above FMN.

The folded hairpin-like structure of L6 that only covers the active site of the other protomer is stabilized by an intramolecular salt bridge network between E291, which usually lies above the flavin cofactor of the adjacent protomer, R294, and D243 (Fig. 6C). In detail, D243 electrostatically holds back R294, which in turn prevents E291 from moving toward the active site of the adjacent protomer.

Unfortunately, it is unclear from the structure how the amino acid substitution induces this semi-self-inhibitory dimer because D283 is not directly involved in stabilizing the folded L6 conformation. A comparison of the protomers in the R283D dimer shows that in protomer B, D283 forms a salt bridge with R343, like in the R283E dimer, whereas in protomer A, D283 is not involved in any interaction and thus free to move (Fig. 6C). We speculate that this freedom of movement translates to the whole loop L6, bringing D243, E291, and R294 together. D283 is located only 3.5 Å away from D243 and thus might push D243 toward R294 via electrostatic repulsion, indirectly supporting the salt bridge network that stabilizes the folded L6 conformation.

R283D also crystallized in its monomeric form (PDB entry: 8S8V) under conditions almost identical to those for the wild-type homodimer. Like in the case of R283E, the monomeric structure of R283D is nearly identical to that of the wild type (RMSD_Cα_ = 0.32 Å), with loop L6 almost entirely missing because of disorder.

Structurally, it is unclear why R283E prefers to crystallize in its monomeric form while R283D prefers the dimeric form. Nevertheless, our crystallographic data show that substituting R283 for an oppositely charged residue generally affects L6 conformation and thus the dimerization process.

#### Dimer formation is independent of residue Y364

Variant Y364P was crystallized in its monomeric (PDB entry: 9EM5) and dimeric (PDB entries: 9EM4 and 9EM6) forms independent of the crystallization additive. Like the other variants, the monomeric form of Y364P is almost identical to the wild-type structure (RMSD_Cα_ = 0.21 Å) and misses nearly the entire loop L6 due to disorder. The sulfate binding site is identical to that in the wild-type homodimer, except for the absence of Y364.

**Figure 7:**
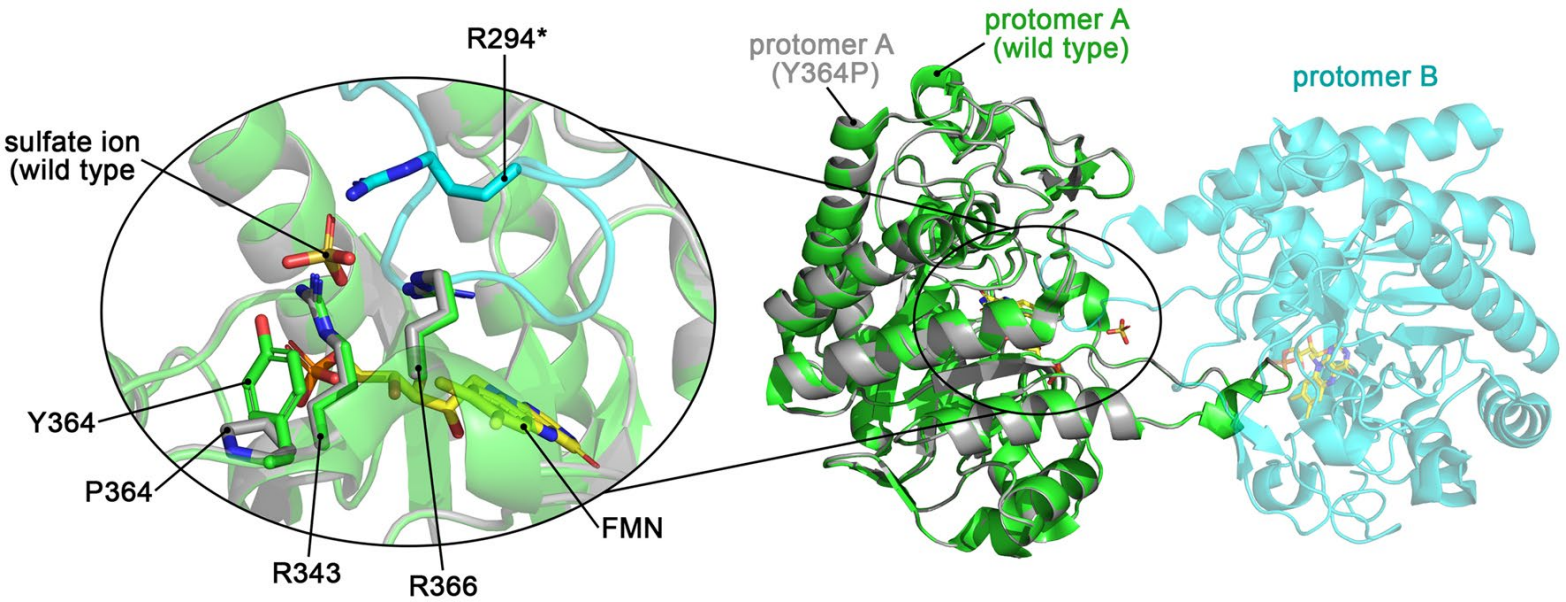
Crystal structure of the Y364P homodimer and comparison with the wild-type homodimer. Protomer A of the Y364P dimer (grey cartoon) is superimposed onto that of the wild-type homodimer (green cartoon). The left inset shows a zoomed view of the sulfate binding site of both homodimers. The amino acid residues forming the site are identical, except for the Y364P substitution.

The self-inhibitory homodimer of Y364P obtained with sulfate (PDB entry: 9EM4) is similar to that of the wild type (RMSD_Cα_ = 3.38 Å; Fig. 7). Both L6 loops adopt the stretched conformation and block the active site of each other's protomer. The slight difference between the dimers arises from the protomers being differently positioned to each other. The sulfate binding sites (R343, Y364/P364, and R366) are the same in both dimers, and despite Y364 being replaced by a proline in Y364P, one protomer in Y364P has a bound sulfate ion at the same position as the wild type, indicating that R343 and R366 are the main binders of the sulfate ion. In the other protomer of Y364P, no sulfate ion is bound. As mentioned before, we previously demonstrated that this sulfate binding site is instead a coenzyme-phosphate binding site responsible for NADPH binding and the enzyme's preference for NADPH over NADH^10^.

The Y364P-homodimer structure obtained without sulfate (PDB entry: 9EM6) is identical to the wild-type homodimer (RMSD_Cα_ = 0.17 Å). Despite missing a tyrosine residue at position 364 and a sulfate ion, *Sl*OPR3 Y364P can still form the same homodimer, clearly positing against the proposed regulation of OPR3 activity by phosphorylation in vivo.

#### *PISA* and further structural analysis of the homodimers

As most of the obtained self-inhibitory dimers differed from each other, we investigated the dimer interfaces of all dimers with PDBePISA^14^. The dimer interfaces differ in the number of H-bonds and salt bridges (Supplementary Table S1). However, in all cases, the strongest contribution to the dimer interface comes from the interactions of E291 with the catalytic residues H185 and H188, except for the semi-inhibitory R283D dimer in which E291 of one protomer interacts with R136 from the other protomer (Table S1).

Besides the PISA-detected differences in the intermolecular (interface) interactions between the dimers, we also observed differences in the intramolecular interactions (i.e., interactions within one protomer) while studying the dimer structures. For example, we did not observe the same set of intramolecular salt bridges within L6 for both protomers in a given homodimer; that is, if L6 of protomer A exhibits the salt bridges X and Y, L6 of protomer B would exhibit at least one different salt bridge Z, indicating that the dimerization is asymmetric with regard to the L6-mediated interactions (see the salt bridges depicted in Fig. 5). Considering the distribution of the charged residues on L6 and the active site entrance and surmising that intramolecular salt bridges—together with H-bonds—dictate the stretched conformation of L6, it is not surprising that salt bridges and/or H-bonds between different residues lead to different L6 conformations and differently positioned and orientated protomers within the homodimer. This ambiguity in the dimers' L6 conformation and compactness (based on their hydrodynamic diameters) supports the structural plasticity of dimerization (Figure S3). These differences among the dimers may explain the peak broadening in some samples during DLS, as multiple dimers of different hydrodynamic diameters were detected.

Taking up that E291-mediated interactions contribute the most to the dimer interface, one can suggest that L6 mimics an OPR3 inhibitor, with residue E291 functioning as its electron-withdrawing group. The large architecture of the active site cavity of OPR3 compared to that of other OPR isozymes is responsible for its broad substrate specificity, making it predestined to accept elongated and/or bulky substrates bearing an electron-withdrawing group such as octadecanoids or even a whole loop^9,15^. Therefore, we doubt that *Sl*OPR3 dimerization has a physiological role in jasmonic acid biosynthesis and believe that homodimerization of *Sl*OPR3 is a rather fortuitous event triggered by high protein concentrations, a large active site cavity, and the fact that L6 mimics a *Sl*OPR3 inhibitor. This would also explain why other OPR isozymes like OPR1, which has a smaller active site cavity, do not dimerize in solution.

It was shown that *Sl*OPR3 variant E291K, which cannot dimerize, has a six-fold faster turnover than the wild type, supporting a regulatory role for the homodimer^9^. However, this increase in the turnover number is rather marginal and mainly indicates a faster FMN reduction in the variant, which is the rate-limiting step in ene-reductases. Furthermore, the faster turnover of E291K does not necessarily have to stem from the variant's inability to dimerize. For example, looking at the previously reported *Sl*OPR3–NADPH_4_ complex structure (PDB entry: 8QMX), position 291 is close to the coenzyme's 2′-phosphate-adenosine tail; therefore, K291 could promote the reductive half-reaction (i.e., reduction of FMN by NADPH) by favorably interacting with the coenzyme^10^.

Nevertheless, a physiological role for the OPR3 self-inhibitory dimer cannot definitively be excluded because other (yet unknown) factors present in a plant cell may promote dimerization and shift the monomer–dimer equilibrium toward the dimer. For example, changes in pH (within cellular compartments), which was not tested in the present study, could trigger OPR3 homodimerization or the crowded environment, particularly in compartments where the jasmonic acid biosynthesis pathways are active, could facilitate OPR3 dimerization by increasing the effective concentration of the enzyme.

### Conclusions

Based on the first crystal structure of *Sl*OPR3 in which the enzyme forms a self-inhibitory dimer, homodimerization was postulated to regulate the enzyme's activity and thus jasmonic acid biosynthesis. As the crystal structure also contained a tyrosine-bound sulfate within the dimer's interface, phosphorylation of this tyrosine (Y364) was suggested to trigger dimerization and, thereby, the autoinhibition of *Sl*OPR3 in vivo.

We showed that the monomer–dimer equilibrium of *Sl*OPR3 is highly dynamic in vitro, as it is sensitive to various factors, including protein concentration, ionic strength, and weakly or even non-binding small-molecule ligands, rendering the dimer relatively weak.

All enzymes (wild type and three variants) could be crystallized in their monomeric and dimeric forms, even variant Y364P, which lacks the putative phosphorylation target Y364. Thus, it seems unlikely that reverse phosphorylation of Y364 serves as a regulation mechanism in vivo.

While our results argue against a phosphorylation-mediated regulation of *Sl*OPR3 dimerization, the enzyme's self-inhibitory dimerization remains a valid mechanism for controlling its activity in vivo. However, our crystallographic data also show that the homodimers, even those of the same enzyme, differ (partially significantly) in their L6 conformation and position and/or orientation of the protomers to each other. Thus, we suggest that dimer formation in *Sl*OPR3 might not be strong enough to be physiologically relevant. Still, a physiological role for the homodimer cannot be entirely rejected based on our findings because other external factors present in a plant cell, such as the crowding effect, could trigger homodimerization and autoinhibition, especially in compartments in which metabolic pathways are active.

As the dynamic nature of *Sl*OPR3 dimerization challenges a straightforward interpretation of a regulatory role for the homodimer, *in*-*vivo* studies are needed to confirm our *in*-*vitro* results and elucidate the molecular basis of *Sl*OPR3 dimerization and its regulation, especially *in planta*.

## Materials and methods

### Protein production

The *Sl*OPR3 variants R283D, R283E, and Y364P were generated by introducing point mutations into the pET21d( +)-6xhis-*Slopr3* plasmid using the Q5® Site-Directed Mutagenesis Kit (New England Biolabs, Ipswich, MA, USA) according to the manufacturer's instructions. Primer pairs were generated using the NEBaseChanger® online tool from New England Biolabs. The presence of the desired mutation was verified by sequencing (LGC Genomics, Berlin, Germany).

All enzymes were recombinantly produced in *E. coli* BL21-CodonPlus (DE3)-RIL cells and purified by Ni–NTA chromatography (Qiagen, Hilden, Germany) as previously described^10^. After Ni–NTA chromatography, the eluted enzymes were rebuffered using a PD-10 column (Cytiva, Freiburg, Germany) in storage buffer: 25 mM NaH_2_PO_4_ (pH 8.0) and 150 mM NaCl. Protein concentrations were determined based on the absorbance at 450 nm using an extinction coefficient of ε_450_ = 12,500 M^-1^ cm^-1^.

### Analytical gel filtration

Analytical gel filtration was performed with a Superdex® 200 Increase 10/300 GL column (Cytiva) coupled to an Äkta explorer system (GE Healthcare, Freiburg, Germany). The storage buffer was used to equilibrate the column and elute the proteins. The molecular mass of the target proteins at a given elution volume was estimated using the Gel Filtration Calibration Kit (mass range: 29–440 kDa, Cytiva) according to the manufacturer's instructions. The same column was used for all protein samples, and 500 µL of each enzyme at a concentration of 2 mg/ml was applied to the column. After each analytical run, further (preparative) runs at higher protein concentrations (10–20 mg/ml) were performed to examine the elution profile with increasing protein concentration.

### Dynamic light scattering (DLS) and HYDROPRO calculations

DLS measurements were performed using a Zetasizer µV (Malvern Instruments Ltd., Malvern, UK) equipped with an 830-nm laser at 25 °C. Before measurement, all samples and buffers were filtered through a 0.2-µm syringe filter. Each enzyme was analyzed at four concentrations (1, 5, 10, and 20 mg/ml) and in the presence and absence of 25 µM ammonium sulfate to study the influence of sulfate (i.e., Y364-bound sulfate mimicking phosphorylation) on the dimerization process.

Reference hydrodynamic diameters for the monomeric and dimeric forms of all enzymes were calculated from the corresponding crystal structures using HYDROPRO software^16^. The following parameters were used: molecular mass, 44.6 and 89.2 kDa for the monomeric and dimeric form, respectively; temperature, 25 °C; solvent viscosity, 0.01 poise; solute partial specific volume, 0.73 cm^3^/g; solution density, 1.0 g/cm^3^.

### Statistical analysis

A one-way ANOVA test was performed to determine whether the addition of ammonium sulfate significantly changed the particle size of *Sl*OPR3 in the DLS experiment. The level of significance was set to *p* < 0.05.

### X-ray crystallography

*Sl*OPR3 wild-type and variant crystals (10 mg/ml) were grown by the vapor-diffusion method using the hanging drop setup. Crystals were grown at 20 °C by equilibrating drops at different protein:reservoir ratios (1:2, 1:1, and 2:1) against 500 µl reservoir solution. To investigate the effect of sulfate on the dimerization process, we crystallized each enzyme in the presence and absence of ammonium sulfate. For the former, the reservoir solution consisted of 0.1 M MES/Tris (pH 6.5), 10–50 mM ammonium sulfate, and 8–16%(*w*/*v*) PEG 8000, while for the latter, it consisted of 0.1 M Tris–HCl (pH 7.5), 50 mM sodium tartrate, and 8–16%(*w*/*v*) PEG 8000. Before data collection, all crystals were cryo-protected in the reservoir solution supplemented with 20% MPD and vitrified in liquid nitrogen.

Diffraction data were collected at DESY (Hamburg, Germany) on beamline P11 and at ESRF (Grenoble, France) on beamline ID30B at 100 K. Data sets were processed and scaled using the XDS package^17^. In some cases, the autoprocessed files available from ESRF were directly used^18^. Data collection information is summarized in Supplementary Tables S2–S5.

All structures were solved by molecular replacement using Phaser from the PHENIX suite^19,20^. A truncated version of PDB entry 8QMX, in which L6 was deleted, was used as the search model. Model building and refinement were performed with Coot and phenix.refine, respectively^21,22^. Final models were validated using MolProbity, and refinement statistics are provided in Supplementary Tables S2–S5^23^. All crystal structures were deposited in the Protein Data Bank (PDB; see Supplementary Tables S2–S5 for PDB entry codes).

### Supplementary Information

Supplementary Information.

## Data Availability

Atomic coordinates and structure factors have been deposited in the Protein Data Bank under accession codes 9EM3, 9EM2, 9EM0, 8S8V, 8QN1, 8QN9, 8S8Y, 9EM5, 9EM6, and 9EM4.
