# Supplementary material for: Analysis of homodimer formation in 12-oxophytodienoate reductase 3 in solutio and crystallo challenges the physiological role of the dimer
Source: Sci Rep. 2024 Aug 5;14:18093. doi: 10.1038/s41598-024-69160-6 (PMC11300593; doi:10.1038/s41598-024-69160-6)
Supplement: Supplementary file 1 — Supplementary Information. [file 41598_2024_69160_MOESM1_ESM.pdf]

**SUPPLEMENTARY INFORMATION FOR**

# Analysis of homodimer formation in 12-oxophytodienoate reductase 3 *in solutio* and *crystallo* challenges the physiological role of the dimer

Bianca Kerschbaumer, Peter Macheroux, Aleksandar Bijelic*

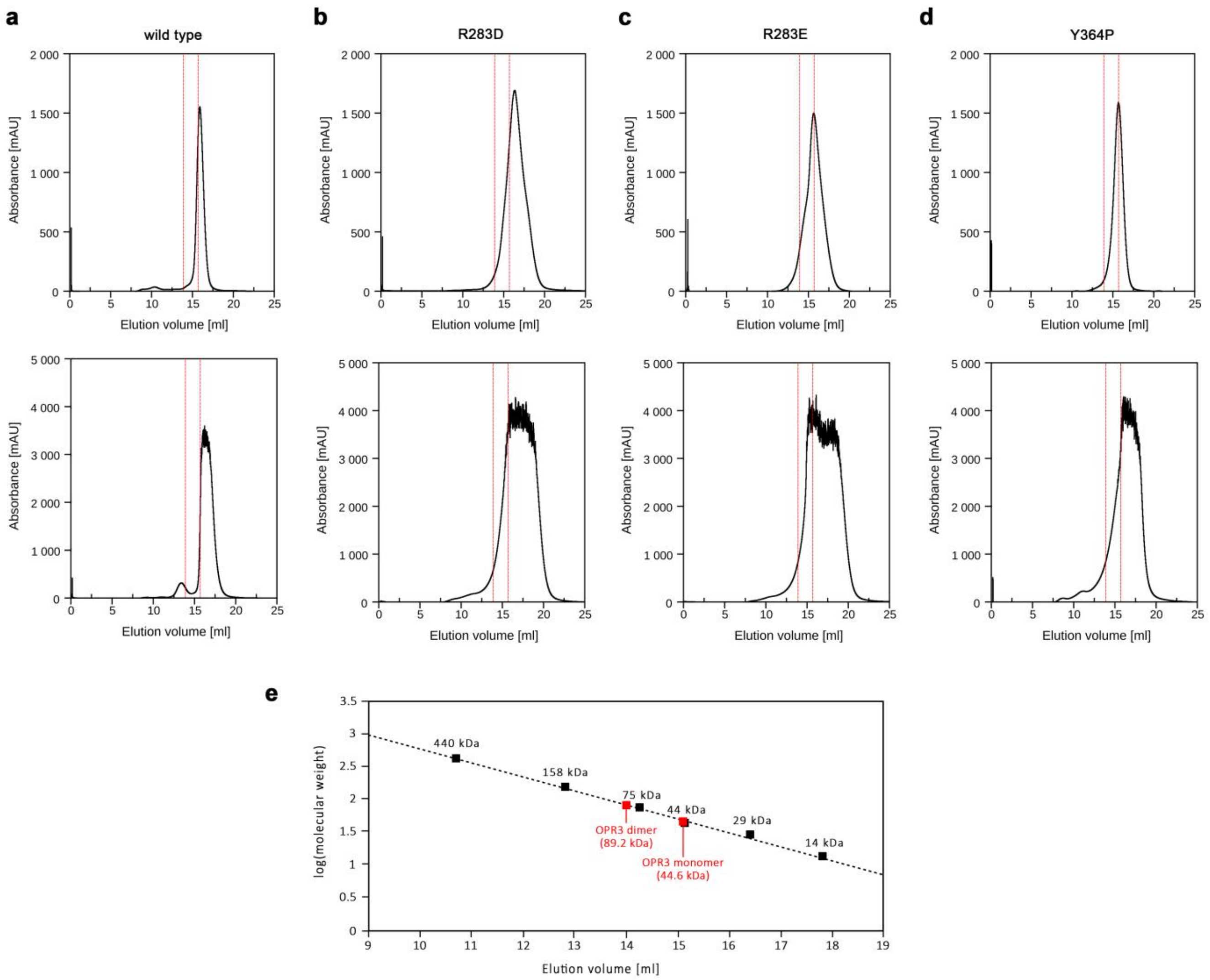


**Supplementary Figure S1.** Analytical gel filtration of *Sl*OPR3 (**a**) wild type, (**b**) R283D, (**c**) R283E, and (**d**) Y364P. The top panels of (**a**)–(**d**) show the chromatograms at a protein concentration of 2 mg/ml while the bottom panels show those at 10–20 mg/ml (overload). The red vertical dashed lines indicate the estimated elution volumes of the enzyme's monomeric (44.6 kDa) and dimeric (89.2 kDa) forms. (**e**) Standard curve generated from a linear fit ($R^2$ = 0.997) of the log molecular weights of the standard proteins (ferritin, 440 kDa; aldolase, 158 kDa; conalbumin, 75 kDa; ovalbumin, 44 kDa; carbonic anhydrase, 29 kDa; ribonuclease A, 14 kDa) versus their elution volume. The (extrapolated) standard curve is shown as a black dotted line. Black boxes indicate data points for generating the standard curve (standard proteins), while red boxes indicate the predicted elution volumes for the monomer and dimer of *Sl*OPR3.

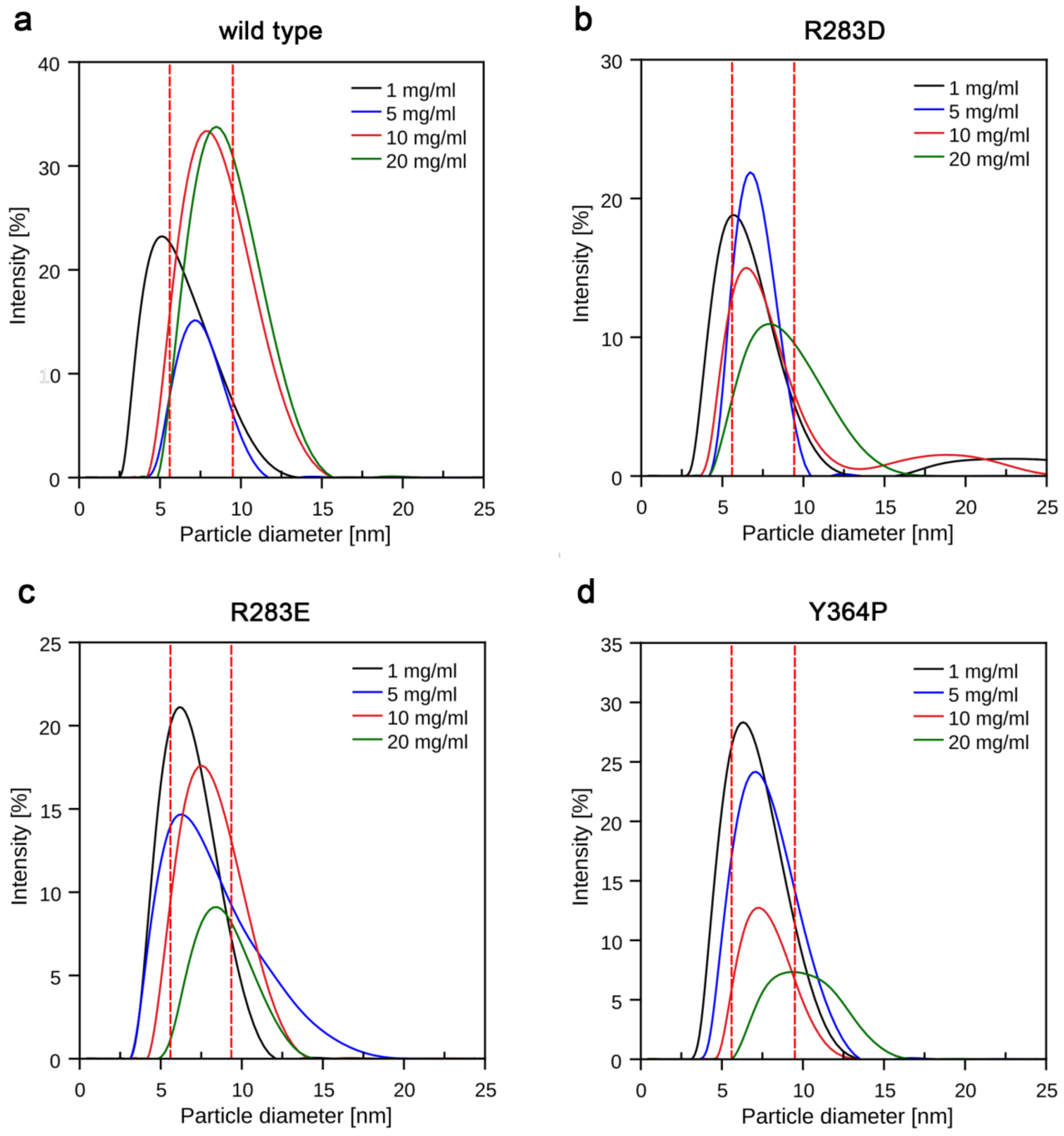


**Supplementary Figure S2.** Dynamic light scattering (DLS) profiles for all enzymes in the presence of 25 mM ammonium sulfate. (**a**) wild type, (**b**) R283D, (**c**) R283E, and (**d**) Y364P. The red vertical dashed lines indicate the calculated particle diameters for the enzymes' monomeric and dimeric states.

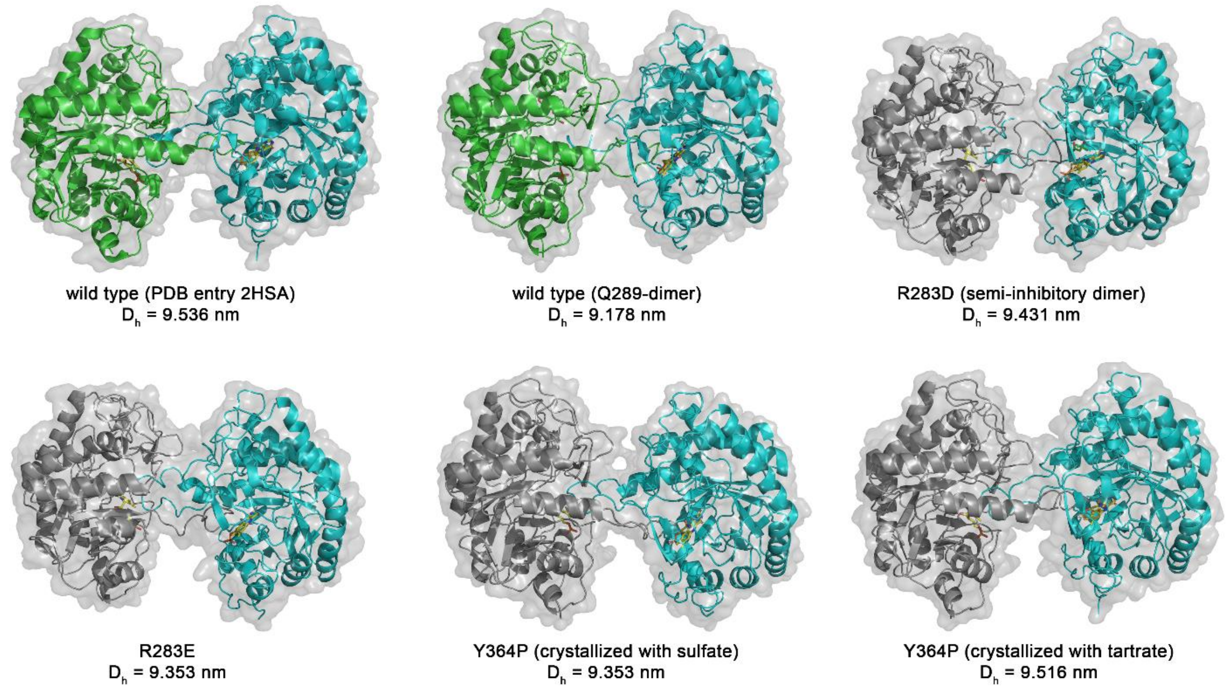


**Supplementary Figure S3.** Structural overview of the *Sl*OPR3 dimers obtained in this study. The hydrodynamic diameter ($D_h$) is given for each dimer. Note the different positions and orientations of the protomers to each other between dimers.

**Supplementary Table S1.** PISA analysis of the homodimer interfaces.

| Enzyme | No. of H-bonds | No. of salt bridges | No. of E291-mediated salt bridges[a] | Interface area (Å$^2$) |
|---|---|---|---|---|
| Wild type | 14 | 11 | 10 | 1489.2 |
| Wild type-Q289[b] | 21 | 15 | 12[b] | 1380.8 |
| R283D | 12 | 6 | 6 | 1169.2 |
| R283E | 13 | 7 | 6 | 1534.8 |
| Y364P | 12 | 8 | 8 | 1367.3 |

[a] This number indicates how many of the total salt bridges include residue E291. Note that PISA assumes that the histidine residues H185 and H188 are charged.

[b] Refers to the novel wild-type dimer reported in this study in which Q289 is located above FMN. Note that E291 of one protomer is not located above FMN in this dimer.

**Supplementary Table S2.** Data collection and refinement statistics.

| | **Wild type monomer** | **Wild type dimer w/o AS[a]** | **Wild type Q289 dimer[b]** |
|---|---|---|---|
| **Data collection** | | | |
| Diffraction source | ESRF, ID30B | ESRF, ID30B | ESRF, ID30B |
| Wavelength (Å) | 0.87 | 0.87 | 0.87 |
| Temperature (K) | 100 | 100 | 100 |
| Detector | Eiger2 X 9M | Eiger2 X 9M | Eiger2 X 9M |
| Space group | $P\ 2_1\ 2_1\ 2$ | $P\ 1\ 2_1\ 1$ | $P\ 1\ 2_1\ 1$ |
| *a*, *b*, *c* (Å) | 87.26, 89.84, 48.88 | 57.85, 89.73, 81.2 | 57.69, 89.66, 80.15 |
| *α*, *β*, *γ* (°) | 90, 90, 90 | 90, 109,17, 90 | 90, 104.17, 90 |
| Total reflections | 147199 (22941) | 954520 (29147) | 346113 (31094) |
| Unique reflections | 48983 (8108) | 136455 (4107) | 102435 (10325) |
| Multiplicity | 3.0 (2.8) | 7.0 (7.1) | 3.4 (3.0) |
| Completeness (%) | 97.49 (97.39) | 99.46 (96.38) | 97.19 (78.01) |
| $R_{pim}$ (%) | 0.25 (1.27) | 0.04 (1.72) | 0.23 (1.71) |
| Mean $I/\sigma(I)$ | 2.72 (0.49) | 9.17 (0.28) | 2.47 (0.38) |
| $CC_{1/2}$ | 0.95 (0.19) | 0.99 (0.13) | 0.96 (0.15) |
| | | | |
| **Refinement** | | | |
| Resolution range (Å) | 42.94–2.27 | 46.67–1.54 | 47.46–1.99 |
| | (2.41–2.27) | (1.56–1.54) | (2.09–1.99) |
| $R_{work}/R_{free}$ (%) | 21.29/26.15 | 17.62/20.01 | 26.33/28.95 |
| RMS (bonds) (Å) | 0.004 | 0.015 | 0.002 |
| RMS (angles) (°) | 0.75 | 1.05 | 0.47 |
| Average *B*-factor ($Å^2$) | 30.21 | 24.98 | 28.85 |
| | | | |
| **Ramachandran plot** | | | |
| Ramachandran favored (%) | 96.37 | 97.06 | 98.12 |
| Ramachandran allowed (%) | 3.63 | 2.94 | 1.88 |
| Ramachandran outlier (%) | 0.00 | 0.00 | 0.00 |
| | | | |
| **PDB ID** | 9EM3 | 9EM2 | 9EM0 |

[a] Unlike the initially reported wild-type homodimer (PDB entry: 2HSA), this homodimer was obtained without (w/o) ammonium sulfate (AS).
[b] Unlike the initially reported wild-type homodimer (PDB entry: 2HSA), this dimer has residue Q289 positioned over FMN.
Values in parenthesis are for the highest resolution bin.

**Supplementary Table S3.** Data collection and refinement statistics.

| | **R283D monomer** | **R283D dimer** |
|---|---|---|
| **Data collection** | | |
| Diffraction source | ESRF, ID30B | DESY, P11 |
| Wavelength (Å) | 0.87 | 1.03 |
| Temperature (K) | 100 | 100 |
| Detector | Eiger2 X 9M | Eiger2 X 16 M |
| Space group | $P\ 2_1\ 2_1\ 2_1$ | $P\ 1\ 2_1\ 1$ |
| *a*, *b*, *c* (Å) | 86.39, 89.31, 95.53 | 81.07 90.78 111.74 |
| *α*, *β*, *γ* (°) | 90, 90, 90 | 90, 109.64, 90 |
| Total reflections | 133610 (8517) | 1048844 (35381) |
| Unique reflections | 37427 (2689) | 150353 (4973) |
| Multiplicity | 3.6 (3.2) | 6.9 (7.0) |
| Completeness (%) | 94.40 (95.57) | 97.94 (97.55) |
| $R_{pim}$ (%) | 0.29 (0.89) | 4.66 (49.65) |
| Mean $I/\sigma(I)$ | 1.71 (0.46) | 9.30 (1.48) |
| $CC_{1/2}$ | 0.90 (0.18) | 0.99 (0.73) |
| | | |
| **Refinement** | | |
| Resolution range (Å) | 44.66–2.69 | 46.28–1.75 |
| | (2.76–2.69) | (1.77–1.75) |
| $R_{work}/R_{free}$ (%) | 26.98/30.73 | 23.35/25.35 |
| RMS (bonds) (Å) | 0.005 | 0.008 |
| RMS (angles) (°) | 1.02 | 0.81 |
| Average *B*-factor (Å$^2$) | 41.27 | 31.03 |
| | | |
| **Ramachandran plot** | | |
| Ramachandran favored (%) | 96.61 | 96.76 |
| Ramachandran allowed (%) | 3.39 | 3.24 |
| Ramachandran outlier (%) | 0.00 | 0.00 |
| | | |
| **PDB ID** | 8S8V | 8QN1 |

Values in parenthesis are for the highest resolution bin.

**Supplementary Table S4.** Data collection and refinement statistics.

| | **R283E monomer** | **R283E dimer** |
|---|---|---|
| **Data collection** | | |
| Diffraction source | DESY, P11 | ESRF, ID30B |
| Wavelength (Å) | 1.03 | 0.87 |
| Temperature (K) | 100 | 100 |
| Detector | Eiger2 X 16 M | Eiger2 X 9M |
| Space group | $P\ 2_1\ 2_1\ 2$ | $P\ 1\ 2_1\ 1$ |
| *a*, *b*, *c* (Å) | 87.68, 89.78, 49.39 | 54.23, 90.36, 78.13 |
| *α*, *β*, *γ* (°) | 90, 90, 90 | 90, 107.97, 90 |
| Total reflections | 315054 (9437) | 477507 (8860) |
| Unique reflections | 48261 (1770) | 137014 (2424) |
| Multiplicity | 6.5 (5.3) | 3.5 (3.7) |
| Completeness (%) | 92.40 (58.57) | 97.58 (45.14) |
| $R_{pim}$ (%) | 3.1 (68.13) | 0.08 (1.41) |
| Mean $I/\sigma(I)$ | 15.41 (1.07) | 5.72 (0.55) |
| $CC_{1/2}$ | 0.99 (0.46) | 0.99 (0.15) |
| | | |
| **Refinement** | | |
| Resolution range (Å) | 43.84–1.6 (1.63–1.6) | 44.80–1.75 (1.78–1.75) |
| $R_{work}/R_{free}$ (%) | 15.27/18.14 | 18.70/21.96 |
| RMS (bonds) (Å) | 0.009 | 0.016 |
| RMS (angles) (°) | 0.99 | 1.37 |
| Average *B*-factor ($Å^2$) | 25.67 | 21.68 |
| | | |
| **Ramachandran plot** | | |
| Ramachandran favored (%) | 98.03 | 96.61 |
| Ramachandran allowed (%) | 1.97 | 3.39 |
| Ramachandran outlier (%) | 0.00 | 0.00 |
| | | |
| **PDB ID** | 8QN9 | 8S8Y |

Values in parenthesis are for the highest resolution bin.

**Supplementary Table S5.** Data collection and refinement statistics.

| | Y364P monomer | Y364P dimer w/o AS[a] | Y364P dimer w AS[b] |
|---|---|---|---|
| **Data collection** | | | |
| Diffraction source | ESRF, ID30B | ESRF, ID30B | ESRF, ID30B |
| Wavelength (Å) | 0.87 | 0.87 | 0.87 |
| Temperature (K) | 100 | 100 | 100 |
| Detector | Eiger2 X 9M | Eiger2 X 9M | Eiger2 X 9M |
| Space group | $P\ 1\ 2_1\ 1$ | $P\ 1\ 2_1\ 1$ | $P\ 1\ 2_1\ 1$ |
| *a*, *b*, *c* (Å) | 44.34, 89.94, 51.28 | 58.01, 90.05, 81.24 | 57.53, 89.64, 80.94 |
| *α*, *β*, *γ* (°) | 90, 105.63, 90 | 90, 109.13, 90 | 90, 109.01, 90 |
| Total reflections | 371217 (14664) | 531956 (6747) | 355661 (17924) |
| Unique reflections | 54069 (14664) | 81190 (1595) | 51254 (2515) |
| Multiplicity | 3.0 (1.0) | 6.6 (4.2) | 6.9 (7.1) |
| Completeness (%) | 98.84 (82.51) | 96.87 (50.48) | 99.26 (87.76) |
| $R_{pim}$ (%) | 0.06 (1.26) | 0.04 (0.98) | 0.05 (0.63) |
| Mean $I/\sigma(I)$ | 5.8 (0.5) | 10.14 (0.57) | 9.09 (0.95) |
| $CC_{1/2}$ | 0.99 (0.35) | 0.99 (0.29) | 0.99 (0.53) |
| | | | |
| **Refinement** | | | |
| Resolution range (Å) | 49.39–1.57 | 46.81–1.72 | 46.5–2.01 |
| | (1.6–1.57) | (1.74–1.72) | (2.05–2.01) |
| $R_{work}/R_{free}$ (%) | 18.69/21.06 | 18.05/21.52 | 20.67/24.41 |
| RMS (bonds) (Å) | 0.017 | 0.009 | 0.014 |
| RMS (angles) (°) | 1.46 | 0.89 | 0.84 |
| Average *B*-factor (Å$^2$) | 19.99 | 29.57 | 38.37 |
| | | | |
| **Ramachandran plot** | | | |
| Ramachandran favored (%) | 97.21 | 97.19 | 96.38 |
| Ramachandran allowed (%) | 2.79 | 2.81 | 3.62 |
| Ramachandran outlier (%) | 0.00 | 0.00 | 0.00 |
| | | | |
| **PDB ID** | 9EM5 | 9EM6 | 9EM4 |

[a] This dimer was crystallized without (w/o) ammonium sulfate (AS).
[b] This dimer was crystallized with (w) ammonium sulfate (AS).
Values in parenthesis are for the highest resolution bin.
